# Supplementary material for: Response to First-Line Treatment with Immune-Checkpoint Inhibitors in Patients with Advanced Cutaneous Squamous Cell Carcinoma: A Multicenter, Retrospective Analysis from the German ADOReg Registry
Source: Cancers (Basel). 2022 Nov 11;14(22):5543. doi: 10.3390/cancers14225543 (PMC9688854; doi:10.3390/cancers14225543)
Supplement: Supplementary file 1 [file cancers-14-05543-s001.zip › cancers-2024661-supplementary.pdf]

Supplementary Tables and Figures

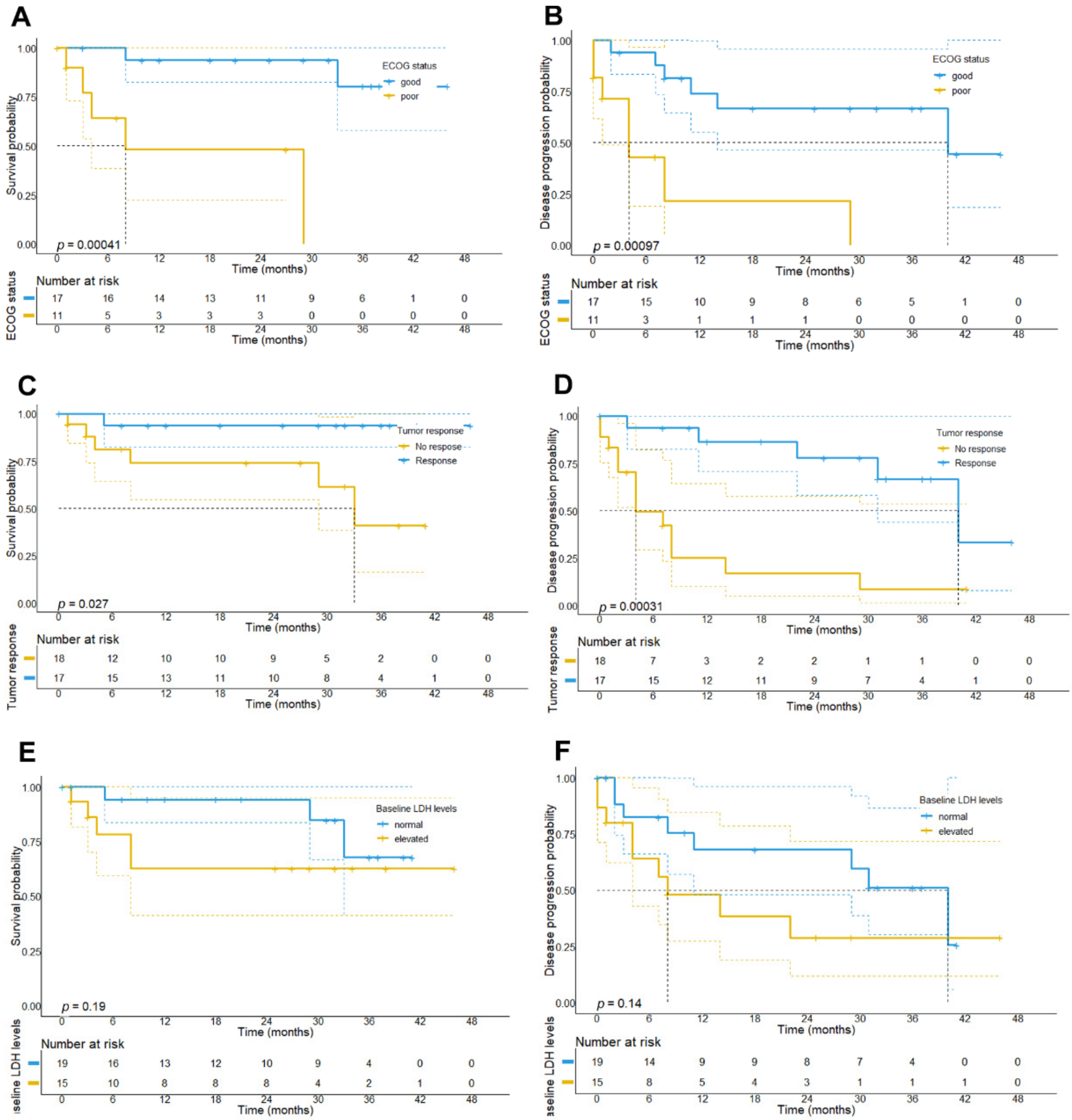

Supplementary Figure S1: Overall survival and Progression-free survival stratified by ECOG performance status (A, B), real-world tumor response (C, D) and baseline serum LDH levels (E, F). Kaplan-Meier plots depicting overall survival rates are shown on the left panel, while plots for progression-free survival are shown on in the subfigures on the right. Results show that a poor ECOG performance status is associated with shorter OS (8.0 vs NR, p<0.001) and PFS (4 vs 40 months, p<0.001). Furthermore, our data reveal that patients achieving complete or partial response upon CPI treatment showed a better OS (median OS: 33.0 months 95% CI: 25.6-40.4 months vs NR, p=0.027) and PFS as compared to patients with stable disease or progressive disease (median PFS: 40 months, 95% CI: 27.0 – 53.0 months vs 4 months, 95% CI: 0-8.5 months, p<0.001). Elevated serum LDH levels at baseline were also associated with a shorter OS and PFS, albeit this association was below statistical significance both for OS (p=0.19) and PFS (40.0 vs 8.0 months, p=0.14).

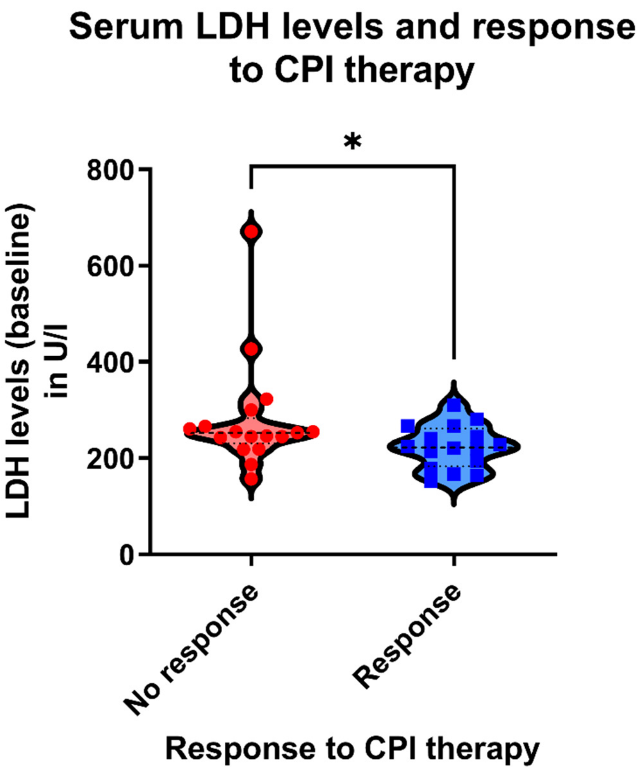

Supplementary Figure S2: Violin plots depicting the association between response to CPI therapy and LDH serum levels at baseline: Patients responding to first-line CPI therapy showed significantly lower LDH levels at baseline (median: 222.5 U/l) as compared to non-responding patients (median: 253.0 U/l, p=0.03).

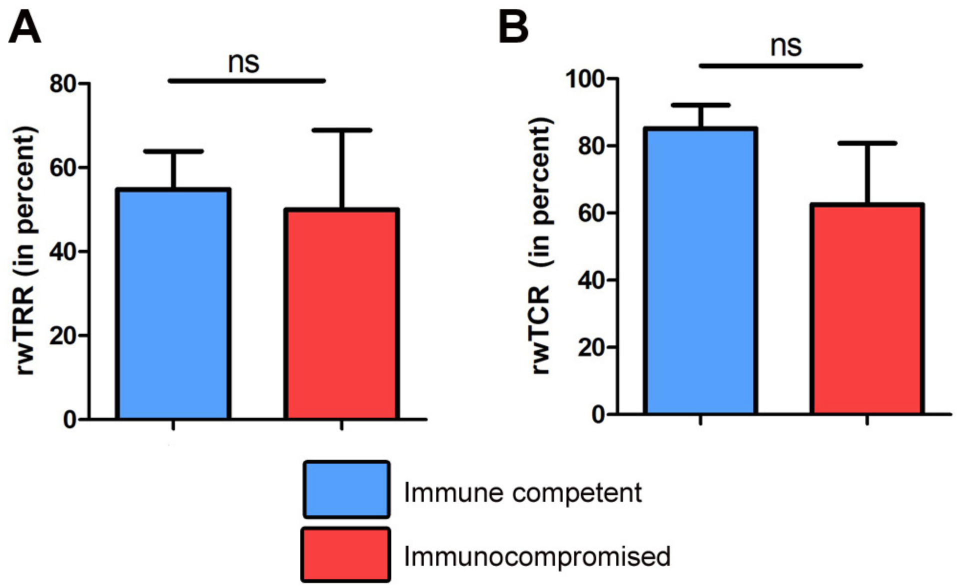

Supplementary Figure S3: Bar charts showing the real-world tumor response rate (rwTRR) (A) and real-world tumor control rate (rwTCR) (B) upon first-line CPI treatment in the overall patient cohort and stratified by the immune status. Although immunocompromised patients showed a somewhat lower response rate to first-line CPI therapy this association was below statistical significance in two-sided Mann-Whitney test.

Supplementary Table S1: Definition of real-world endpoints used in this study.

Endpoint	Outcome
**Primary**	
Overall survival (OS)	The time interval from start of first-line CPI treatment to date of death. Patients alive at the date of last contact were censored.
**Secondary**	
Real-world tumor response (rwTR)	Best tumor response was defined as complete response (CR), partial response (PR), stable disease (SD), or progressive disease (PD) according real-world response assessments1. (The best therapy response using both the clinical assessments in the medical record and the radiological assessment in the staging findings are captured within the ADOReg database).
Real-world tumor response rate (rwTRR)	The proportion of patients with a complete response or partial response based on real-world response assessments# relative to all patients initiating treatment.
Real-world tumor control rate (rwTCR)	The proportion of patients who had a complete response, partial response, or stable disease based on real-world response assessments
Progression-free survival (PFS)	The time interval from start of first-line CPI treatments to physician-reported date of progression, death date or start date of a new treatment due to progression of disease (whichever event occurred first). Patients without a progression event or date of death were censored at the date of last contact.
Treatment-related adverse events	Adverse events during CPI therapy that were treated as clinically indicated and retrospectively graded according to the Common Terminology Criteria for Adverse Events, version 5.0 (25). Only AEs ≥grade II were evaluated because low grade AEs may not be documented thoroughly in routine clinical practice.

[1] Complete response: complete resolution of all visible disease; partial response: disease still present, with partial reduction in size of visible disease in some or all areas without any areas of increase in visible disease; stable disease: no change in overall size of visible disease or mixed response.; progressive disease: substantial increase in the overall size of all visible tumor lesions or occurrence of new tumor lesions.

Supplementary Table S2: Univariate Cox proportional hazards model for overall survival.

Variables	Subgroups	HR	95% CI	*p*-value
Age (years)	>79 vs ≤79	2.16	0.54-8.63	0.28
Gender	Female vs male	0.70	0.086-5.73	0.74
Primary tumor site	Head-neck vs other	1.70	0.35-8.23	0.51
Immunosuppression	Yes vs no	8.96	2.07-38.85	**0.003**
LDH at baseline	Elevated vs normal	2.52	0.60-10.60	0.206
ECOG status at baseline	Poor performance status (>1) vs good performance status (<=1)	19.27	2.13-174.7	**0.009**
Horizontal tumor diameter (cm)	≥2cm vs <2cm	1.23	0.11-13.64	0.87
Vertical tumor thickness (mm)	>6mm vs ≤6mm	2.64	0.58-12.0	0.21
Poor pathological differentiation	No vs Yes	0.206	0.03-2.13	0.206
Distant metastasis at advanced stage	Yes vs No	1.03	02.1-5.10	0.974
Prior RTx treatment	Yes vs no	1.97	0.24-16.1	0.53
In-sano resection	Yes vs no	0.554	0.066-4.63	0.59
CPI treatment duration	≥5 months vs <5months	0.55	0.138-2.21	**0.04**
rwTR to 1L CPI treatment	CR vs PR vs SD vs PD	0.28	0.10-0.78	**0.015**

The p value is indicated in bold numbers when statistically significant. Abbreviations: CPI= checkpoint inhibitor(s); CR = complete response, ECOG= eastern cooperative oncology group; LDH= lactate dehydrogenase; PR = partial response; rwTR=real-world tumor response; SD = stable disease, PD = Progressive disease, RTx= radiotherapy; rwTR = real-world tumor response, HR = hazard ratio, CI = confidence interval; 1L = first-line.

Supplementary Table S3: Univariate Cox proportional hazards model for progression-free survival.

Variables	Subgroups	HR	95% CI	*p*-value
Age (years)	>79 vs ≤79	1.74	0.68-4.44	0.24
Gender	Female vs male	0.62	0.14-2.74	0.53
Primary tumor site	Head-neck vs other	0.89	0.33-2.39	0.81
Immunosuppression	Yes vs no	2.55	0.92-7.11	0.073
LDH at baseline	Elevated vs normal	2.05	0.77-5.41	0.15
ECOG status at baseline	Poor performance status (>1) vs good performance status (<=1)	6.10	1.82-20.34	**0.003**
Horizontal tumor diameter (cm)	>2cm vs <2cm	5.29	0.32-86.93	0.243
Vertical tumor thickness (mm)	>6mm vs ≤6mm	0.40	0.11-1.49	0.17
Poor pathological differentiation	Yes vs no	3.27	0.83-12.82	0.09
Distant metastasis at advanced stage	Yes vs No	0.63	0.18-2.20	0.47
Prior RTx treatment	Yes vs no	1.27	0.36-4.46	0.71
In-sano resection	No vs Yes	2.02	0.67-6.11	0.21
CPI treatment duration	>5 months vs <5months	0.34	0.12-0.97	**0.04**
rwTR to 1L CPI treatment	CR vs PR vs SD vs PD	0.27	0.14-0.53	**<0.001**

The p value is indicated in bold numbers when statistically significant. Abbreviations: 1L = first-line; CPI= checkpoint inhibitor(s); CR = complete response, ECOG= eastern cooperative oncology group; LDH= lactate dehydrogenase; PR = partial response; rwTR=real-world tumor response; SD = stable disease, PD = Progressive disease, RTx= radiotherapy; rwTR = real-world tumor response, HR = hazard ratio, CI = confidence interval.

Supplementary Table S4. Response to immune checkpoint inhibitor therapy stratified by the class of the immune status of the patients.

Outcome	Immunocompetent	Immunocompromised	Fisher´s exact Test
Real-world tumor response - no. (%)			0.093
Progressive disease (PD)	4 (13.3%)	3 (33.3%)	
Stable disease (SD)	10 (33.3%)	1 (11.1%)	
Partial response (PR)	6 (20.0%)	4 (44.4%)	
Complete response (CR)	7 (23.3%)	0	
Could not be evaluated	3 (10.0%)	1 (11.1%)	
Real-world tumor response rate [1]			1.0
No. (%)	13 (48.1%)	4 (50.0%)	
95% CI [3]	31.9-71.3%	15.7-84.3%	
Real-world tumor control rate [2]			0.321
No. (%)	23 (85.2%)	5 (62.5%)	
95% CI [3]	66.3-95.8%	24.5-91.5%	
Progress upon CPI therapy			
No. (%)	12 (40.0%)	6 (66.7%)	0.255
95% CI [3]	22.7-69.4%	29.9-92.5%	

Abbreviations: [1,2] Real-world tumor response rate was defined as the percentage of patients who obtained CR or PR; Real-world tumor control rate was defined as the percentage of patients who obtained CR, PR, or SD. Both rwTRR and rwTCR were calculated based on the total number of patients with known tumor response. [3] The 95% confidence intervals were calculated using the Clopper–Pearson method.
